# Yipishen Xiezhuo Jiedu Decoction in Ameliorating Kidney Damage Through miR-223/NLRP3/Caspase-1 Pathway

**DOI:** 10.2174/0118715303402744250704071034

**Published:** 2025-07-10

**Authors:** Jianfei Weng, Dengyong Zheng, Huijun Chen, Zhangcheng Huang, Xiaojing Wu, Weijie Zheng, Zi Yu, Qinghui Xu

**Affiliations:** 1 Department of Urology, The Second People’s Hospital Affiliated to Fujian University of Traditional Chinese Medicine, Fuzhou, China;; 2 Department of Nephrology, The Second People’s Hospital Affiliated to Fujian University of Traditional Chinese Medicine, Fuzhou, China;; 3 Department of Urology, Fujian University of Traditional Chinese Medicine, Fuzhou, Fujian, 350100, China

**Keywords:** Hyperuricemic nephropathy, kidney disease, YPSXZJDD, inflammation, miR-223, NLRP3

## Abstract

**Introduction:**

Hyperuricemia Nephropathy (HN) is an emerging metabolic disorder that predisposes individuals to Chronic Kidney Disease (CKD), yet effective treatments remain limited. Inflammation plays a pivotal role in HN-induced kidney injury, with the NLRP3 inflammasome serving as a central mediator of this process. This study investigates the therapeutic effects of Yipishen Xiezhuo Jiedu Decoction (YPSXZJDD), a traditional Chinese medicine, on HN-induced kidney injury through the miR-223/NLRP3/Caspase-1 pathway.

**Materials and Methods:**

The key active components of YPSXZJDD were screened using UHPLC-Q Exactive Orbitrap-MS, and a Protein-Protein Interaction (PPI) network diagram was constructed to explore potential mechanisms of action. The identified components were then utilized to intervene in both cellular and animal models of hyperuricemic nephropathy, evaluating their therapeutic effects and underlying mechanisms.

**Results:**

Catalpol and Tanshinone IIA were identified as the key active components of YPSXZJDD. These compounds significantly mitigated renal epithelial cell apoptosis and inflammation by upregulating miR-223, which in turn inhibited the NLRP3/Caspase-1 pathway. The upregulation of miR-223 led to a marked reduction in NLRP3 activity and inflammatory responses, thereby alleviating HN-induced kidney damage.

**Discussion:**

The findings of this study underscore the critical role of miR-223 in regulating the NLRP3 inflammasome and highlight its potential as a therapeutic target for HN. The inhibition of the NLRP3/Caspase-1 pathway by miR-223 significantly reduces inflammation and renal injury, demonstrating the therapeutic efficacy of YPSXZJDD. These results offer a novel perspective on the application of traditional Chinese medicine in treating HN, highlighting the importance of miR-223 in regulating inflammation.

**Conclusion:**

This study demonstrates that YPSXZJDD alleviates HN-induced kidney injury by upregulating miR-223 and inhibiting the NLRP3/Caspase-1 pathway. The therapeutic potential of YPSXZJDD is supported by its ability to mitigate inflammation and renal damage, offering a promising approach for HN treatment. Further research into the broader role of miR-223 in kidney disease and related conditions is warranted to expand the understanding of its therapeutic applications.

## 1 INTRODUCTION

The global burden of Chronic Kidney Disease (CKD) is steadily escalating, with Hyperuricemic Nephropathy (HN) increasingly recognized as a prominent etiological factor [1, 2]. The rising incidence of HN closely mirrors shifts in modern dietary and lifestyle patterns [3]. Historically attributed to urate crystal deposition within renal tissues, which triggers inflammation, HN is now understood to involve a spectrum of crystal-independent mechanisms. These include endothelial dysfunction, heightened oxidative stress, Renin-Angiotensin System (RAS) activation, Epithelial-to-Mesenchymal Transition (EMT) in tubular epithelium, and vascular smooth muscle proliferation [4]. Despite the routine application of urate-lowering agents combined with symptomatic management, current therapeutic outcomes remain suboptimal [5]. Encouragingly, traditional herbal medicine offers a versatile armamentarium for HN management, particularly through multi-component formulations with broad pharmacological profiles [6].

Ren *et al*. identified Pectolinoside (PEC), a flavonoid compound, as a potent nephroprotective agent in a murine HN model, where it suppressed the TGF-β1/SMAD3/STAT3 signaling cascade, reduced systemic uric acid levels, and alleviated inflammation and fibrotic progression [2]. Similarly, Shui *et al*. demonstrated that the traditional Si-Miao Pill effectively inhibited EMT and restrained NLRP3 inflammasome activation in a chronic HN mouse model, yielding therapeutic effects comparable to febuxostat [7]. The present study focuses on YPSXZJDD, a polyherbal formulation incorporating *Astragalus membranaceus, Smilax glabra, Dioscorea tokoro* Makino, *Pyrrosia lingua, Rheum palmatum, Cornus officinalis, Dioscorea opposita, Lysimachia christinae, Atractylodes macrocephala*, and *Salvia miltiorrhiza.* These constituents are traditionally believed to tonify kidney yang, reinforce essence, and enrich the blood. Among them, *Atractylodis Macrocephalae* notably contributes to qi regulation and phlegm dispersion. Contemporary investigations support the clinical utility of such formulations in mitigating not only HN but also broader metabolic and inflammatory renal conditions [8, 9], marking a convergence between empirical tradition and molecularly informed therapy.

Two pharmacologically active constituents—catalpol and tanshinone IIA—derived from Chinese medicinal herbs have garnered significant attention for their renoprotective capabilities. Renal injury, particularly in the context of CKD and diabetic nephropathy, is driven by oxidative stress, chronic inflammation, and fibrotic remodeling. The early stages are typically characterized by oxidative and inflammatory damage, whereas irreversible fibrotic progression underlies the functional decline. Catalpol has demonstrated strong antioxidative properties through ROS scavenging and attenuation of oxidative injury [10]. It also suppresses inflammatory cytokine expression, including TNF-α and IL-6, thereby reducing renal inflammatory burden [11]. Moreover, catalpol helps preserve nephron integrity by safeguarding tubular and interstitial architecture [12]. Tanshinone IIA exerts comparable antioxidative and anti-inflammatory effects, with additional antifibrotic activity evidenced by reduced collagen deposition and downregulation of fibrosis-associated mediators [12]. These combined actions position both agents as promising candidates for intervention in chronic and metabolic renal disorders.

A growing body of research has also illuminated the role of microRNA-223 (miR-223) in modulating inflammation under hyperuricemic conditions [13, 14]. This discovery has opened new investigative pathways in traditional Chinese medicine, wherein miRNA-targeted approaches are explored as potential anti-inflammatory strategies. Although miRNAs are short (~23 nucleotides), non-coding RNAs with still-evolving functional understanding, they are increasingly recognized for their regulatory influence over differentiation, metabolism, and immune response [15]. Clinical and experimental data indicate a downregulation of miR-223 in individuals with gout, a pattern that normalizes post-remission [16]. Mouse models deficient in miR-223 exhibit intensified MSU-induced joint inflammation, with elevated inflammatory cell infiltration and cytokine release [17]. Mechanistically, miR-223 targets NLRP3 mRNA in both MSU-induced rats and fibroblast-like synoviocytes, thereby modulating pyroptosis and inflammatory signaling [17]. Collectively, these findings underscore miR-223's pivotal role in controlling NLRP3-dependent pathways and, by extension, in the pathophysiology of urate-associated disorders. Therefore, it is proposed that enhancing miR-223 expression to regulate inflammatory cascades may offer a novel and mechanistically grounded therapeutic strategy for HN—one that shifts the treatment paradigm from symptom relief to molecular modulation.

## 2 MATERIALS AND METHODS

### 2.1 Study Design

This study is an experimental pharmacological study combining network pharmacology, cell-based assays, and an animal model to explore the therapeutic mechanism of YPSXZJDD in HN. The experimental framework included *in vitro* testing using HK-2 human renal tubular epithelial cells and *in vivo* validation in a rat model of HN.

In the animal experiment, twenty Specific-Pathogen-Free (SPF) grade male Sprague-Dawley (SD) rats, aged 6–8 weeks and weighing approximately 220 grams, were used. Animals were randomly assigned to four groups (n = 5 per group):

1. Control group
2. Model group (HN model)
3. YPSXZJDD treatment group (10 g/kg/day)
4. YPSXZJDD + miR-223 inhibitor group

The hyperuricemia nephropathy model was induced by oral gavage of adenine (0.1 g/kg) and potassium oxonate (1.5 g/kg) for 3 weeks, followed by a 14-day treatment intervention. To reduce potential bias, grouping and treatment were performed randomly, and outcome assessments (*e.g.*, histology, ELISA, and qPCR) were conducted by investigators who were blinded to group assignments.

The sample size (n = 5 per group) was based on previous studies involving similar renal injury models and was deemed sufficient to detect medium-to-large effect sizes with acceptable variability. All procedures were approved by the Institutional Animal Care and Use Committee (IACUC) of the Second People's Hospital Affiliated to Fujian University of Traditional Chinese Medicine (Approval No. FJPSPH-IAEC2024112), and ethical considerations regarding animal welfare were strictly followed.

Data were collected through standardized methods, including:

UHPLC-Q Exactive Orbitrap-MS for compound profiling;

 Flow cytometry for apoptosis detection;

 qRT-PCR and Western blot for molecular expression;

 ELISA for inflammatory markers. 

 Histopathological and Immunohistochemical (IHC) staining were performed for renal injury assessment, and statistical analysis was conducted using GraphPad Prism 8.0. Normality was tested using the Kolmogorov–Smirnov or Shapiro–Wilk test. For normally distributed data, one-way ANOVA with Dunnett’s or Bonferroni’s post hoc test was used. For non-normally distributed data, appropriate non-parametric tests were applied. Statistical significance was considered at *P* < 0.05.

### 2.2 Materials and Reagents

The custom formulation YPSXZJDD includes the following components. *Astragalus membranaceus* (30g), *Smilax glabra* (40g), *Dioscorea tokoro* Makino (12g), *Pyrrosia lingua* (12g), *Rheum palmatum* (5g), *Cornus officinalis* (12g), *Dioscorea opposita* (20g), *Lysimachia christinae* (20g), *Atractylodes macrocephala* (10g), and *Salvia miltiorrhiza* (15g). All herbs were sourced from Shanghai Yuanye Biotechnology Co., Ltd. (Shanghai, China). CCK-8, Enzyme-Linked Immunosorbent Assay (ELISA), Serum Creatinine (SCr), and Blood Urea Nitrogen (BUN) assay kits were obtained from Beyotime Biotechnology Co., Ltd. Antibodies for Western blot targeting NLRP3, Caspase-1, Gasdermin D (GSDMD), and Glyceraldehyde-3-phosphate dehydrogenase (GAPDH) were supplied by Servicebio Technology Co., Ltd. (Wuhan, China). Catalpol and Tanshinone IIA, used as additional analytical standards, were acquired from Selleck Biotech Co., Ltd. (Japan).

### 2.3 UHPLC-Q Exactive   Orbitrap-MS   Analysis   of  YPSXZJDD

UHPLC-Q Exactive Orbitrap-MS analysis of YPSXZJDD was performed in order to obtain the main active ingredients in the formulation. The data acquisition utilized a UPLC system (Vanquish, Thermo, USA) coupled with a high-resolution mass spectrometer (Q Exactive HFX, Thermo, USA). Chromatographic separation was performed using a Waters HSS T3 column (100 × 2.1 mm, 1.8 μm). The mobile phases included ultrapure water with 0.1% formic acid (Phase A) and acetonitrile with 0.1% formic acid (Phase B). The optimized chromatographic conditions were as follows. A flow rate of 0.3 mL/minute, a column temperature of 40°C, and an injection volume of 2 μL.

The gradient elution program was as follows: 0–1 min, 100% A; 1–12 minutes, a gradual shift to 5% A; 12–13 minutes, 5% A; 13.1–17 minutes, re-equilibration at 100% A. Samples were maintained at 4°C in the autosampler during analysis. To ensure reliable results, samples were analyzed in a randomized order, with QC samples included periodically to monitor system stability [18].

### 2.4 Network Pharmacological Analysis of the Targets of Action of the Key Components of YPSXZJDD on HN

The chemical structures of active compounds were retrieved from the PubChem database (https://pubchem.ncbi. nlm.nih.gov/). To predict potential target proteins, a pharmacophore-based approach was employed using the PHNrmMapper tool (http://lilab-ecust.cn/pHNrmmapper/). This sophisticated reverse pharmacophore matching tool provided detailed target information, including target names and corresponding scores, which reflect the affinity strength between compounds and targets. Higher scores signified a more substantial binding potential, and only compounds with target scores exceeding 3 were selected for further analysis, ensuring a focus on the most relevant interactions.

To validate the reliability of the predicted targets, the UniProt database (http://www.uniprot.org/) was utilized. This validation step ensured that the identified targets aligned with experimentally validated protein data, enhancing the credibility of these findings. By integrating these validated targets, a comprehensive and high-quality database of active component targets was established, laying the groundwork for downstream network pharmacology studies. Throughout the process, meticulous attention was given to refining and cross-checking data, ensuring that the results were robust and meaningful for subsequent analyses.

### 2.5 Identification of YPSXZJDD Compounds and Functional Target Prediction

The functional targets of YPSXZJDD’s primary active compounds were predicted using the Bioinformatics Analysis Tool for Molecular Mechanism of Traditional Chinese Medicine (BATMAN-TCM) platform (http://bionet.ncpsb. org.cn/batman-tcm/), a bioinformatics tool specifically designed to uncover the molecular mechanisms underlying Traditional Chinese Medicine (TCM). This platform facilitates the identification of herb-component-target interactions, leveraging advanced algorithms to associate bioactive compounds with their probable functional targets.

The prediction process involves assigning interaction scores, which quantify the strength of the relationship between each compound and its potential targets. These scores serve as a critical benchmark for prioritizing targets. In this study, a “score cutoff” of 5.785 was established to ensure the selection of robust interactions. Additionally, an adjusted *P*-value threshold of 0.05 was applied to maximize the coverage of biologically meaningful targets while maintaining statistical rigor.

Through this approach, the active compounds were systematically mapped to their corresponding targets, enabling a deeper exploration of the therapeutic mechanisms of YPSXZJDD. This rigorous and data-driven strategy not only enhances the reliability of the predicted targets but also provides a solid foundation for subsequent functional and pathway enrichment analyses.

### 2.6 Identification of Potential HN-Related Targets

To comprehensively identify molecular targets associated with HN, a systematic search was conducted using the keyword “hyperuricemic nephropathy” across multiple authoritative databases, including OMIM, GeneCards, CTD, DisGeNET, HPO, DrugBank, and NCBI-Gene. These databases provided insights into genetic associations, disease mechanisms, and pharmacological targets related to HN. Additionally, a detailed review of clinical guidelines and literature on HN diagnosis and treatment was performed to gather data on therapeutic drugs and their molecular mechanisms. This integrated approach enabled the compilation of a robust target dataset, encompassing genetic, pharmacological, and clinical information, laying the foundation for future research and therapeutic advancements in HN management.

### 2.7 Identification of Overlapping Targets and Protein-Protein Interaction (PPI) Analysis

A Venn diagram was constructed using the Venny platform, enabling the identification of overlapping targets between YPSXZJDD compounds and Hyperuricemic Nephropathy (HN). These intersections were highlighted as potential therapeutic targets for YPSXZJDD in the treatment of HN, offering critical insights into its mechanism of action.

For further exploration of the functional interactions between these targets, a PPI analysis was performed using STRING (https://string-db.org/), a comprehensive tool for examining protein associations based on experimental data, text mining, and computational predictions. The analysis was restricted to “Homo sapiens” to ensure relevance to human biology, with a minimum interaction confidence score set at 0.500 to prioritize high-confidence interactions.

The resulting PPI network was systematically organized, and the data was exported in a .tsv file format. This dataset served as a foundational resource for subsequent analyses, facilitating the identification of key nodes and pathways involved in the therapeutic effects of YPSXZJDD. By leveraging these advanced computational tools, the study ensures a robust and detailed understanding of target interactions, paving the way for deeper mechanistic insights.

### 2.8 Screening and Evaluation of Principal Targets and Bioactive Compounds

For screening and evaluating the primary targets and bioactive compounds, data from the STRING analysis were imported into Cytoscape 3.8.2 (https://cytoscape.org/). A Protein-Protein Interaction (PPI) network was first constructed, using the median values of degree, closeness, and betweenness centrality to identify the core targets. The “VLOOKUP” function was then applied to systematically map these core targets to YPSXZJDD components that met the specified criteria. This process facilitated the construction of a comprehensive “YPSXZJDD-compound-core target-disease” interaction network, elucidating the relationships between the bioactive compounds and key disease-associated targets.

### 2.9 Gene Ontology (GO) and Kyoto Encyclopedia of Genomes (KEGG) Analysis of Key Target Pathways of YPSXZJDD

To clarify the biological processes linked to core targets across various clusters and their involvement in signal transduction, Gene Ontology (GO) and Kyoto Encyclopedia of Genes and Genomes (KEGG) pathway enrichment analyses were carried out using the Metascape platform (https://metascape.org). This tailored analysis involved inputting a list of target gene names and applying a minimum gene overlap criterion of three, a significance threshold of *P* < 0.01, and a minimum enrichment factor of 1.5 The top 20 enriched terms were then visually represented in a bubble chart, generated through an online bioinformatics visualization tool (http://www.bioinformatics.com.cn/), to facilitate a more intuitive understanding of the data.

### 2.10 Cell Culture and Intervention

Human renal epithelial HK-2 cells were procured from Procell Life Science & Technology Co., Ltd. (Wuhan, China). Cultivation was performed in DMEM/F12 medium, enriched with 8% Fetal Bovine Serum (FBS), and maintained at 37°C within a 5% CO_2_ atmosphere. HK-2 cells were seeded at a density of 1.5 × 10^5 cells/mL, using 0.1 mL or 1 mL per well in 96-well or 6-well plates, respectively. Post 12 hours of serum starvation in 0.2% FBS, the HK-2 cells were exposed to Uric Acid (UA) at a concentration of 20 mg/dL for 36 hours. Concurrently, treatments with Catalpol and Tanshinone IIA were administered at concentrations of 10 nM, 25 nM, and 50 nM for 36 hours.

### 2.11 Cell Viability

For the cell viability assay, HK-2 cells were initially plated in 96-well plates following the previously described protocol. To each well, 10 μL of Cell Counting Kit-8 (CCK-8) solution was added, and the plates were incubated at 37°C for one hour. After incubation, absorbance was recorded at 450 nm to determine cell viability [19].

### 2.12 Apoptosis Detection

Apoptosis in HK-2 cells was evaluated using an Annexin V-Fluorescein Isothiocyanate (FITC) apoptosis detection kit (Beyotime, China). After trypsin digestion, cells were collected by centrifugation and resuspended in binding buffer. They were then incubated with Annexin V-FITC at room temperature for 15 minutes, and apoptosis levels were analyzed *via* flow cytometry [20].

### 2.13 Western Blotting

Total cellular proteins were extracted using RIPA lysis buffer (1:1000) (040-483, KeyGen Biotech, Nanjing, China), and the resulting supernatant was then collected. Protein concentration was quantified by Bicinchoninic Acid (BCA) assay (Thermo Fisher Scientific, China). Extracted proteins were separated by SDS-PAGE and transferred to a PVDF membrane. After blocking with 5% BSA (A55865, Thermo Fisher Scientific, China), the membrane was incubated overnight at 4°C with NLRP3, Caspase-1, GSDMD, and GAPDH primary antibodies (ab263899, ab286125, ab219800, Abcam, China). The secondary antibody was incubated with HRP-coupled secondary antibody, and protein bands were detected by Enhanced Chemiluminescence (ECL) and observed using the ChemiDoc™XRS imaging system (Bio-Rad Laboratories, USA).

### 2.14 Quantification of RNA Expression *via* qRT-PCR

Total RNA was isolated using the Trizol method, ensuring high-quality RNA extraction. The purity and concentration of the RNA samples were determined using a NanoDrop Micro Nucleic Acid Assay instrument to ensure suitability for downstream applications. Complementary DNA (cDNA) was synthesized in accordance with the manufacturer's protocol provided in the reverse transcription kit (Takara, Tokyo, Japan).

Quantitative Real-Time PCR (qRT-PCR) was performed using SYBR Green Mix (Takara) on an ABI 7300 Real-Time PCR system (Applied Biosystems, Foster City, CA, USA). Each reaction was conducted in triplicate to ensure accuracy and reproducibility. Data were analyzed using the 2−ΔΔCt method to quantify relative gene expression levels. The specific primers used for amplification were as follows: miR-223 forward primer, GCAGTGTTACGCTCCGTGTA, and reverse primer, CATGAGCCACACTTGGGGTA. This meticulous approach ensured the reliability of the gene expression data generated.

### 2.15 Enzyme-Linked Immunosorbent Assay (ELISA)

IL-1β concentrations in cell supernatants and mouse serum samples were quantified using a commercially available ELISA kit (Abcam, China). Samples were carefully collected and prepared to minimize variability. The assay was performed following the manufacturer’s detailed protocol to ensure accuracy and consistency. Absorbance readings were obtained using a microplate reader, and IL-1β levels were calculated based on the standard curve generated during the assay. All measurements were performed in triplicate to enhance the reliability and reproducibility of the results.

### 2.16 Dual-Luciferase Reporter Gene Assay

The binding interaction between miR-223 and the NLRP3 3' Untranslated Region (UTR) was computationally predicted using starBase (http://www.starbase.sysu.edu.cn/). Based on the predictions, sequences corresponding to the wild-type (wt-NLRP3) and a mutated version (mut-NLRP3) of the binding site were designed and synthesized. These sequences were subsequently cloned into dual-luciferase reporter plasmids.

Cells were transfected with either the wt or mut constructs alongside 50 nM of miR-223 mimic or mimic-NC, followed by incubation for 48 hours. After incubation, cell lysates were prepared using diluted passive lysis buffer. The luciferase activity of each sample was measured using the Luciferase Assay System, with firefly luciferase serving as the reporter and Renilla luciferase as the internal control. The reaction was terminated by adding 1X Stop & Glo^®^ reagent, allowing precise quantification of the relative fluorescence intensity. All experiments were conducted in triplicate to ensure data reliability [21].

### 2.17 HN Rat Model Construction and Treatment

SD rats, 6-8 weeks, males weighing around 220 grams, were provided by Beijing Vital River Laboratory Animal Technology Co., Ltd.. Rats were housed under controlled conditions at 24°C with 65 ± 15% humidity and a 12-hour light/dark cycle. They were provided standard laboratory chow and water and allowed one week of acclimatization before any experimental procedures.

Twenty rats were randomly allocated into four groups: control, model, model + YPSXZJDD (10 g/kg), and model + YPSXZJDD (10 g/kg) + miR-223 inhibitor, with n=5 per group. A rat model of hyperuricemia-induced acute kidney injury was established by daily gavage of an adenine (0.1 g/kg) and potassium oxybate (1.5 g/kg) solution for three weeks. Following the induction period, the model + YPSXZJDD group received YPSXZJDD by gavage, while the control and model groups received an equivalent volume of CMC-Na. The model + YPSXZJDD + miR-223 inhibitor group was administered both YPSXZJDD and a miR-223 inhibitor (RiboBio, Guangzhou, China) for 14 days. At the study’s conclusion, all rats were anesthetized by intraperitoneal injection of sodium pentobarbital (50 mg/kg body weight) to ensure minimal pain and distress, monitored by reflex absence and lack of response to stimuli. Once deeply anesthetized, the rats were humanely euthanized through cervical dislocation, in line with the Animal Euthanasia Guidelines set forth by the Medical Ethics Committee of The Second People’s Hospital Affiliated to Fujian University of Traditional Chinese Medicine. All animal experiments in this study were approved by the Institutional Animal Care and Use Committee (IACUC) of the Second People's Hospital Affiliated to Fujian University of Traditional Chinese Medicine (Approval No. FJPSPH-IAEC2024112), and were conducted by the National Institutes of Health Guide for the Care and Use of Laboratory Animals (8th edition, 2011).

### 2.18 Biochemical Analysis

A the treatment period, each rat from the experimental groups was housed individually in metabolic cages for a 24hour period, facilitating the collection of both urine and blood samples under controlled conditions. Blood samples were drawn *via* the tail vein and immediately centrifuged at 3500 rpm for 15 minutes at 4°C to separate the serum. Serum biochemical parameters, including Serum Uric Acid (SUA), Serum Creatinine (SCr), and Blood Urea Nitrogen (BUN), were measured using a fully automated biochemical analyzer for precise quantification. Similarly, urine samples were collected over the 24 hours, and the levels of Urinary Uric Acid (UUA) and Urinary Creatinine (UCr) were also analyzed using the same biochemical system. The Fractional Excretion of Uric Acid (FEUA), an indicator of renal uric acid handling, was calculated based on the formula:

FEUA = SUA × UCr UUA × SCr

### 2.19 Histological Examination

Kidney tissues were preserved in 4% paraformaldehyde and then embedded in paraffin. Sections, each 4 μm thick, were meticulously sliced from the paraffin blocks. A subset of these sections was stained with Hematoxylin and Eosin (H&E) for subsequent histopathological analysis.

### 2.20 Immunohistochemistry (IHC)

Tissues were preserved in formalin, followed by dehydration and paraffin embedding. The sections were incubated with primary antibodies overnight at 4°C. On the following day, the sections were incubated with HRP-conjugated secondary antibodies for 1 hour at 37°C. Post-incubation, the sections underwent washing with PBS and distilled water, then were treated with freshly prepared DAB solution (diaminobenzidine) for visualization purposes [22].

### 2.21 Statistical Analysis

Data analysis was conducted using GraphPad Prism 8.0, with each experiment repeated in triplicate. Normality of the data was evaluated using the Kolmogorov-Smirnov or Shapiro-Wilk tests. For data following a normal distribution, group comparisons were performed using One-Way Analysis Of Variance (ANOVA) with Dunnett’s or Bonferroni’s post hoc tests. For data not meeting normality assumptions, non-parametric tests were applied. Statistical significance was set at a *P*-value of < 0.05.

## 3 RESULTS

### 3.1 Metabolomic Analysis  of  Key   Components   in   YPSXZJDD

**Fig. (1):**
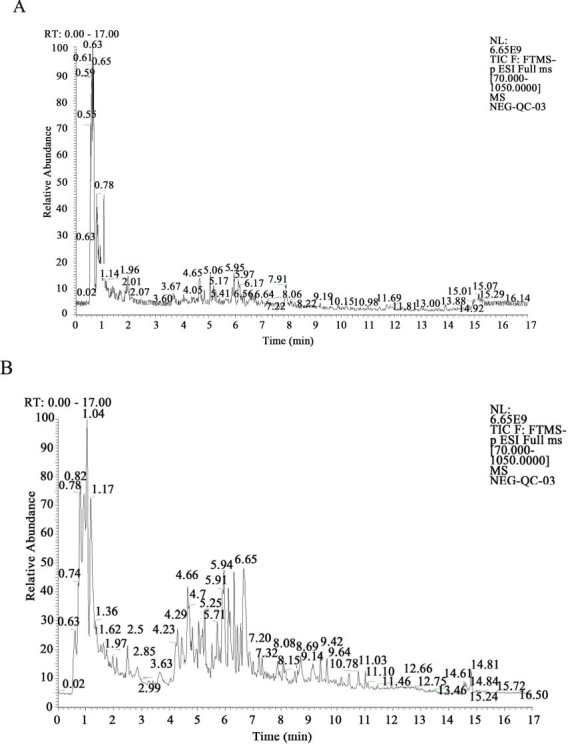
Total ion flow diagrams of YPSXZJDD in positive ion mode and negative ion mode: A: positive ion mode; B: negative ion mode.

The positive and negative ion modes of the advanced UHPLC-Q Exactive Orbitrap-MS technique were utilized. By calculating the relative abundance values of the compounds, 12 major components were identified with relative abundance values of ≥1 in all three YPSXZJDD samples (Fig. **1**, Table **1**). Furthermore, selected catalpol and tanshinone IIA with the highest abundance values for subsequent experiments.

### 3.2 YPSXZJDD Modulates the miR-223/NLRP3 Axis and Inhibits Pyroptosis-related Protein Activation in HN

**Fig. (2):**
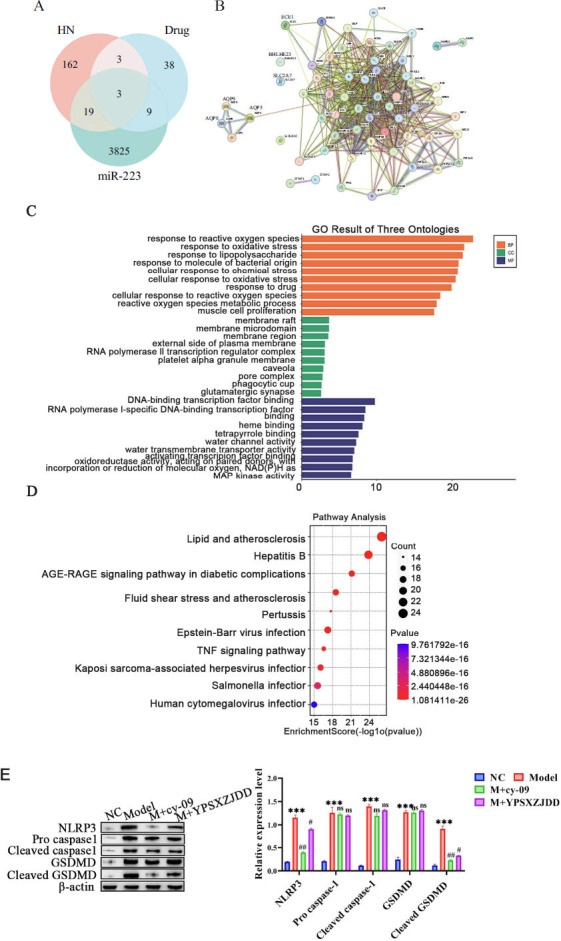
YPSXZJDD inhibits NLRP3 inflammasome activation *via* the miR-223 axis in hyperuricemic nephropathy. (**A**) Venn diagram showing three overlapping genes among HN-related genes, YPSXZJDD drug targets, and miR-223 predicted targets.; (**B**) Protein–Protein Interaction (PPI) network constructed from the intersecting targets using the STRING database. Core hub genes (*e.g.*, NLRP3, CASP1, PTGS2) show extensive interactions, while AQP family members form a distinct subnetwork.; (**C**) GO enrichment analysis of the intersecting genes, highlighting biological processes (BP), Cellular Components (CC), and Molecular Functions (MF) enriched among the targets. (**D**) KEGG pathway enrichment analysis showing significant involvement in the AGE-RAGE signaling pathway, lipid metabolism, TNF signaling, and various infection-related pathways. (**E**) Western blot analysis of NLRP3, pro-caspase-1, cleaved caspase-1, GSDMD, and cleaved GSDMD protein expression in kidney tissues from different groups (NC: normal control, Model: HN model group, M+cy-09: model + miR-223 activator, M+YPSXZJDD: model + YPSXZJDD treatment). The right panel shows quantitative analysis of protein expression normalized to β-actin. Data are expressed as mean ± SD, with n = 3 independent biological replicates per group. Statistical analysis was performed using one-way ANOVA with Bonferroni’s post hoc test. Statistical significance: ***p < 0.001 *vs* NC, ^##^*p* < 0.01 *vs* Model, ns: not significant.

To elucidate the molecular basis of YPSXZJDD’s renoprotective action, an integrative network pharmacology approach was employed, incorporating HN-associated genes, predicted targets of YPSXZJDD, and miR-223-regulated candidates (Fig. **2A**). This tri-layer analysis revealed three overlapping genes, implicating them as potential downstream effectors of miR-223 and as shared mediators in both HN pathogenesis and the therapeutic response to YPSXZJDD. PPI mapping *via* the STRING database uncovered a highly interconnected subnetwork centered on inflammation- and apoptosis-related molecules, notably NLRP3, PTGS2, BAX, CASP1, and GSDMD, indicating their possible cooperative roles in inflammasome assembly and pyroptosis. Additionally, a discrete module consisting of AQP3, AQP5, and AQP9 suggests functional engagement in water channel regulation and renal osmoregulation (Fig. **2B**). GO enrichment pinpointed biological processes such as oxidative stress response, lipopolysaccharide signaling, and cellular adaptation to chemical stimuli—hallmarks of renal inflammation (Fig. **2C**). KEGG pathway analysis highlighted significant enrichment in AGE–RAGE signaling in diabetic complications, lipid metabolism, and atherosclerosis, as well as the TNF pathway (Fig. **2D**). Notably, the AGE–RAGE axis exhibited the most significant enrichment (*p* = 1.08 × 10^−26^), emphasizing the centrality of oxidative-inflammatory stress in disease progression. To experimentally corroborate the predicted involvement of the NLRP3 inflammasome, protein expression levels of NLRP3, pro- and cleaved caspase-1, and GSDMD (both full-length and cleaved forms) in renal tissues were assessed utilizingWestern blotting (Fig. **2E**). YPSXZJDD administration significantly attenuated the expression of NLRP3, cleaved caspase-1, and cleaved GSDMD (*p* < 0.001), whereas levels of pro-caspase-1 and full-length GSDMD remained unchanged. These findings indicated effective inhibition of inflammasome activation and subsequent pyroptosis. Notably, the extent of suppression was comparable to that observed with CY-09, a known miR-223 agonist, suggesting that YPSXZJDD mitigates renal inflammation at least in part through miR-223-driven blockade of the NLRP3/caspase-1/GSDMD pathway.

### 3.3 Protective Effects of YPSXZJDD Key Components on Uric Acid-Induced Renal Tubular Epithelial Cell Injury

**Fig. (3):**
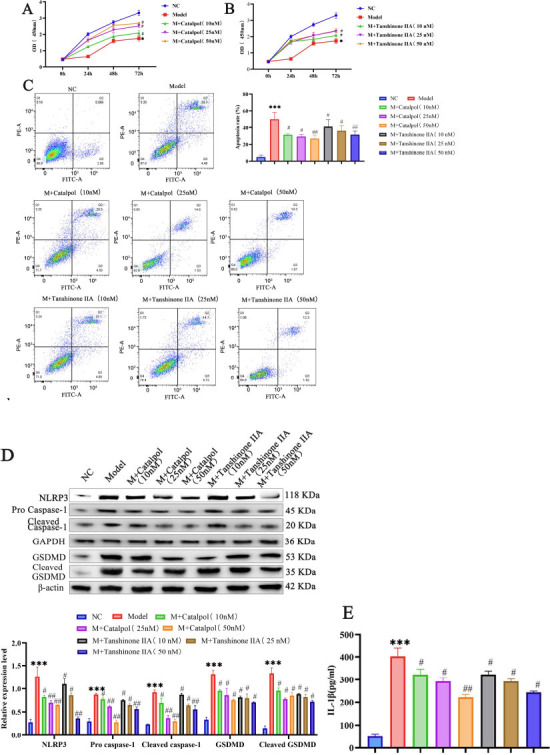
Protective effects of catalpol and tanshinone IIA on UA-induced renal cell injury. (**A**) CCK8 assay results showing the effect of Catalpol on HK2 cell viability; (**B**) CCK8 assay results indicating the impact of Tanshinone IIA on HK-2 cell viability; (**C**) Flow cytometry analysis demonstrating cell apoptosis rates across treatment groups; (**D**) Western blot analysis illustrating the expression levels of NLRP3, Caspase-1, and GSDMD proteins; (**E**) ELISA analysis quantifying IL-1β levels in cell supernatants. Data are presented as mean ± SD, based on three independent biological replicates (n = 3 per group). Statistical analysis was performed using one-way ANOVA followed by Bonferroni’s post hoc test. Statistical significance is denoted as follows: *P < 0.05, **P < 0.01, ***P < 0.001 compared to the NC group; ^#^*P* < 0.05, ^##^*P* < 0.01, ^###^P < 0.001 compared to the Model group.

To investigate the cytoprotective effects of catalpol and tanshinone IIA on Uric Acid (UA)-induced injury in human renal tubular epithelial cells, HK-2 cells were treated with 10, 25, or 50 nM of each compound. CCK-8 assays showed that UA significantly impaired cell viability over time compared to the normal control group (***p < 0.001), while catalpol and tanshinone IIA treatment restored viability in a dose- and time-dependent manner (Figs. **3A**, **B**). Statistically significant improvements were observed at all concentrations (#*p* < 0.05, ##*p* < 0.01 *vs*. model group). Flow cytometry demonstrated a substantial increase in apoptosis in UA-treated cells (***p < 0.001 *vs*. NC), which was significantly reduced by catalpol and tanshinone IIA treatment (^#^*p* < 0.05, ^##^*p* < 0.01), particularly at 50 nM (Fig. **3C**). Western blot analysis revealed that UA exposure induced marked upregulation of NLRP3, cleaved caspase-1, and cleaved GSDMD (***p < 0.001 *vs*. NC), while pro-caspase-1 and full-length GSDMD levels were not significantly altered. Both catalpol and tanshinone IIA downregulated these pyroptosis-related proteins in a concentration-dependent fashion (^#^*p* < 0.05, ^##^*p* < 0.01 *vs*. model group), as shown in Fig. (**3D**). Consistent with these findings, IL-1β levels in the cell supernatant were significantly elevated in the UA model group (***p < 0.001 *vs*. NC) and were substantially suppressed by catalpol and tanshinone IIA treatment (^#^*p* < 0.05, ^##^*p* < 0.01 *vs*. model; Fig. **3E**). These results collectively indicated that catalpol and tanshinone IIA alleviate UA-induced injury by suppressing NLRP3 inflammasome activation and downstream pyroptotic signaling.

### 3.4 Targeting of miR-223 by YPSXZJDD Key Components and its Effect on the NLRP3/Caspase-1 Axis

**Fig. (4):**
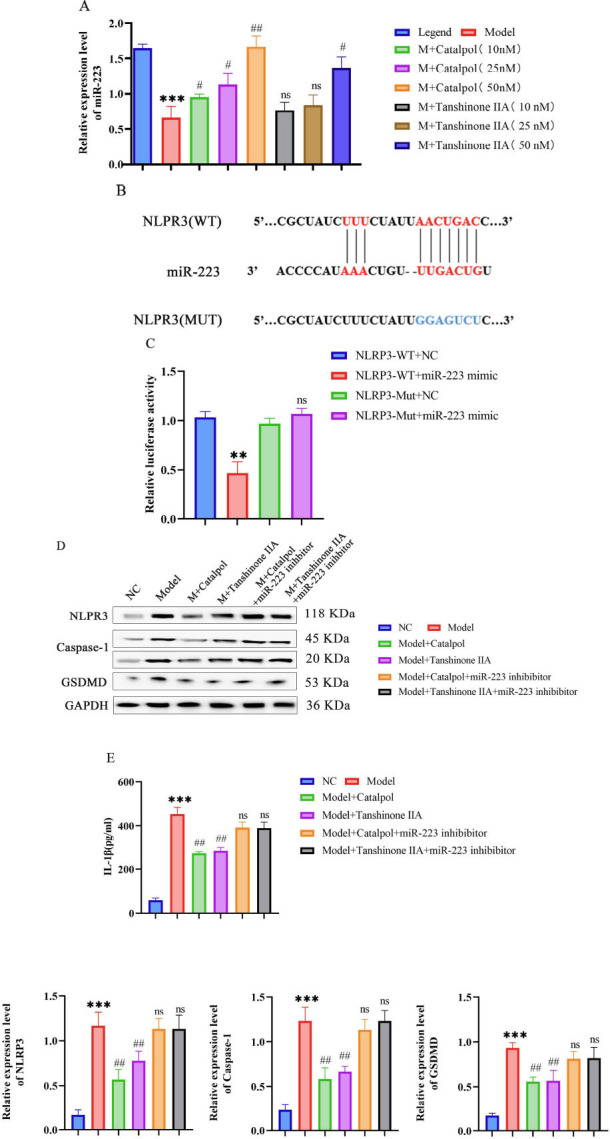
miR-223 Regulation by Catalpol and Tanshinone IIA in NLRP3 Pathway Modulation. (**A**) RT-qPCR analysis demonstrating miR-223 expression levels across treatment groups; (**B**) Predicted binding sequence between NLRP3 and miR-223, showing potential regulatory interaction sites; (**C**) Dual-luciferase reporter gene assay results confirming direct interaction between miR-223 and NLRP3, indicated by reduced luciferase activity in the WT group upon miR-223 introduction, with no significant effect in the Mut group; (**D**) Western blot analysis showing relative expression levels of NLRP3, Caspase-1, and GSDMD proteins across treatment conditions; (**E**) ELISA analysis of IL-1β levels in cell supernatants, illustrating changes in inflammatory marker expression following treatment. All data are expressed as mean ± SD, based on three independent biological replicates (n = 3 per group). Statistical analysis was performed using one-way ANOVA followed by Bonferroni’s post hoc test. Significance is indicated as follows: *P < 0.001 *vs* NC; ^#^*P* < 0.05, ^##^*P* < 0.01, ^###^*P* < 0.001 *vs* Model; ns: not significant.

To further elucidate the mechanism by which catalpol and tanshinone IIA exert their anti-inflammatory effects, the regulatory role of miR-223 in NLRP3 inflammasome signaling was examined. Quantitative PCR showed a significant downregulation of miR-223 in the model group compared to the standard control (***p < 0.001), which was dose-dependently reversed by catalpol and tanshinone IIA treatment. Maximal restoration was observed at 50 nM, indicating concentration-dependent efficacy (Fig. **4A**). Target prediction analysis revealed a conserved miR-223 binding site within the 3’ Untranslated Region (UTR) of NLRP3 mRNA (Fig. **4B**). Dual-luciferase assays confirmed direct binding as luciferase activity was significantly suppressed in cells transfected with the wild-type NLRP3 3’UTR and miR-223 mimic (**p < 0.01), while the mutant construct abolished this interaction (Fig. **4C**). Western blot analysis further validated the downstream impact of miR-223 modulation. Protein levels of NLRP3, cleaved caspase-1, and cleaved GSDMD were significantly reduced following catalpol or tanshinone IIA treatment (***p < 0.001), whereas pro-caspase-1 and full-length GSDMD remained unaffected (Fig. **4D**). These effects were abolished upon co-treatment with a miR-223 inhibitor, indicating a miR-223-dependent regulatory mechanism. In parallel, ELISA revealed elevated IL-1β levels in the model group, which were significantly decreased by catalpol and tanshinone IIA (^##^*p* < 0.01 *vs*. model). Again, this anti-inflammatory effect was reversed upon miR-223 inhibition (Fig. **4E**). Together, these results indicate that catalpol and tanshinone IIA modulate the miR-223/NLRP3/caspase-1/GSDMD axis to suppress inflammasome activation and pyroptosis in the context of renal injury.

### 3.5 YPSXZJDD Alleviates Renal Injury in HN Mice Through the miR-223/NLRP3/Caspase-1 Axis

**Fig. (5):**
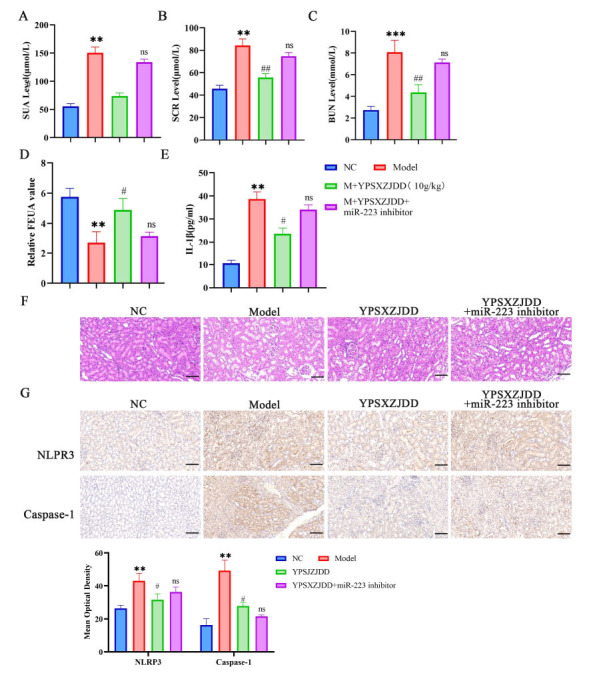
YPSXZJDD Alleviates Renal Injury in HN Mice. (**A**) YPSXZJDD treatment significantly reduces SUA levels; (**B**) Reduction in SCr levels following YPSXZJDD treatment; (**C**) YPSXZJDD lowers BUN levels, indicating improved kidney function; (**D**) Increase in FEUA with YPSXZJDD treatment.; (**E**) ELISA analysis shows decreased IL-1β levels in the YPSXZJDD group, reflecting reduced inflammation; (**F**) H&E staining of kidney tissue shows histopathological changes, with a scale bar of 0.1mm, highlighting tissue integrity improvements with YPSXZJDD; (**G**) IHC analysis of NLRP3 and Caspase-1 expression levels in kidney tissues, with a scale bar of 0.1mm, demonstrating YPSXZJDD's effect on reducing inflammation markers. Data are shown as mean ± SD, based on three independent biological replicates (n = 3 per group). Statistical analysis was performed using one-way ANOVA followed by Bonferroni’s post hoc test. Significance indicators: *P < 0.001 *vs* NC; ^#^*P* < 0.05, ^##^*P* < 0.01, ^###^*P* < 0.001 *vs* Model; ns: not significant.

Based on the results from the cell experiments, the therapeutic effects of YPSXZJDD in an HN mouse model were further evaluated. Following drug intervention, the model group exhibited significant increases in serum SUA, SCr,

 BUN, and IL-1β levels compared to the control group, while FEUA was decreased. In contrast, the treatment group receiving YPSXZJDD showed an opposite trend in these parameters, indicating an ameliorative effect. However, the addition of a miR-223 inhibitor inhibited the therapeutic effects of YPSXZJDD (Figs **5A**-**E**). Histopathological analysis of kidney tissues revealed that the control group had healthy, reddish tissue, while the model group displayed extensive white areas indicative of tissue damage, with inflammatory cell infiltration and disrupted renal architecture, including dilatation and vacuolization. Treatment with YPSXZJDD partially restored the tissue structure, but the presence of the miR-223 inhibitor again hindered tissue repair (Fig. **5F**). IHC analysis further demonstrated that the expression of NLRP3 and Caspase-1 was upregulated in the model group, whereas YPSXZJDD effectively reduced their expression (Fig. **5G**).

## 4 DISCUSSION

A growing body of evidence indicates that HN is an independent risk factor for renal injury, contributing significantly to kidney damage and associated mortality. Nevertheless, the scarcity of effective therapeutic interventions underscores the pressing need for targeted strategies to mitigate HN-related renal dysfunction [23]. Recent investigations have highlighted the pivotal role of inflammation, particularly the NLRP3 inflammasome, in the pathogenesis of HN-induced renal injury [24]. HN promotes the oligomerization of the NLRP3 inflammasome and subsequent caspase-1 activation, which facilitates the maturation of pro-IL-1β into IL-1β and amplifies downstream inflammatory cascades [7]. Urate crystals, abundant in HN, are potent activators of NLRP3, fostering inflammasome assembly and the release of pro-inflammatory cytokines, thereby exacerbating tissue damage and immune cell recruitment [25, 26]. Consequently, the NLRP3-Caspase-1 axis is currently regarded as a viable therapeutic target for anti-inflammatory strategies in HN.

In the present study, characterization of the primary phytochemical constituents of YPSXZJDD was performed using advanced UHPLC-Q-TOF/MS analysis, which identified catalpol and tanshinone IIA as putative key bioactive compounds. Comprehensive analysis suggested that catalpol and tanshinone IIA are the principal bioactive constituents underpinning the formulation's core therapeutic effects. Application of these identified substances to uric acid-challenged HK-2 cells and in HN animal models directly administered with YPSXZJDD markedly attenuated inflammatory responses and cellular damage. This furnishes initial evidence 

supporting their role in ameliorating the HN-associated inflammatory milieu. While these findings posit catalpol and tanshinone IIA as principal bioactive components of YPSXZJDD, based on their relative abundance and established pharmacological properties, it is also acknowledged that the polyherbal nature of YPSXZJDD implies a broader spectrum of bioactive interactions. The therapeutic efficacy of traditional Chinese medicine formulations frequently stems not from a singular dominant compound but from the synergistic interplay of multiple constituents. Several other compounds identified *via* UHPLC-Q Orbitrap-MS, including those derived from Astragali Radix, are recognized for their nephroprotective capabilities, attributed to their capacity to bolster antioxidant defenses and modulate immune function [27]. Similarly, Salviae Miltiorrhizae Radix et Rhizoma is acknowledged for its ability to mitigate renal fibrosis and injury, primarily through anti-inflammatory actions and enhancement of microcirculatory dynamics [28, 29]. These pharmacological insights contribute to validating the traditional therapeutic claims of YPSXZJDD with contemporary scientific evidence. Although catalpol and tanshinone IIA may serve as principal effectors, these other constituents might support or potentiate their activities through network-level mechanisms. In future investigations, it is intended to systematically evaluate potential synergistic interactions among secondary compounds, possibly employing combinatorial index analysis, omics-based correlation modeling, or multi-compound co-treatment trials, to better elucidate the holistic pharmacodynamics of YPSXZJDD.

Intersection of predicted miR-223 targets with YPSXZJDD-responsive genes and HN-associated genes revealed a refined cohort of overlapping candidate genes. These genes were further corroborated through protein-protein interaction analysis, functional enrichment (GO and KEGG), and experimental validation, thereby establishing miR-223 as a central mechanistic node linking herbal therapy in HN to inflammasome regulation. While the role of miR-223 in attenuating NLRP3 inflammasome activation is well-documented across various inflammatory conditions, this investigation is the first to demonstrate this regulatory axis within the context of YPSXZJDD treatment for HN. Given the dysregulated expression of miR-223 in HN and its regulatory impact on NLRP3 [30], it is posited that the therapeutic efficacy of catalpol and tanshinone IIA against HN might be mediated *via* the miR-223/NLRP3 axis. Exploration of the regulatory framework of miR-223 and its interplay with other pathways affecting NLRP3 inflammasome activity revealed that diminished miR-223 expression directly correlated with NLRP3 upregulation, thereby exacerbating inflammatory responses. Conversely, YPSXZJDD administration restored miR-223 expression. Functional assessments using a miR-223 inhibitor confirmed that miR-223 inhibition abrogated the protective effects of YPSXZJDD and its active components (catalpol and tanshinone IIA), thereby establishing a causal role for miR-223 in modulating the NLRP3/Caspase-1/GSDMD axis. Furthermore, dual-luciferase reporter assays directly validated the binding of miR-223 to the 3′UTR of NLRP3, furnishing mechanistic evidence of post-transcriptional repression. Although the importance of miR-223 has been demonstrated using inhibitor-based assays, the absence of direct genetic modulation (such as miR-223 overexpression or knockdown) and the lack of rescue experiments combining miR-223 inhibition with an NLRP3 inhibitor limit the strength of causal inference. These additional functional validations would help confirm whether miR-223 directly regulates NLRP3 and mediates the protective effects of YPSXZJDD. Future studies will incorporate such approaches to better elucidate the mechanistic link between miR-223 and the NLRP3 inflammasome axis.

To investigate upstream regulatory dynamics, a bioinformatic analysis indicated that transcription factors such as NF-κB and STAT3—all established regulators of miR-223 transcription—are potentially modulated by compounds within YPSXZJDD, including quercetin and baicalin. Although experimental validation of these transcriptional modulators is ongoing, these findings suggest that YPSXZJDD exerts its effects through a multi-tiered regulatory network operating at both transcriptional and post-transcriptional levels. This integrated mechanism further elucidates how traditional herbal formulations can precisely modulate inflammatory pathways and lays the groundwork for future investigations involving rescue experiments or transcription factor modulation.

While this study employed miR-223 inhibitors to demonstrate the functional reliance of the NLRP3 inflammatory response on miR-223, it is acknowledged that direct genetic manipulations, such as miR-223 gene knockout or overexpression in transgenic models, would furnish more definitive confirmation of causality. Such assays were beyond the scope and technical capacity of the present study. Nonetheless, the collective evidence from inhibitor-based loss-of-function studies, dual-luciferase assays, and downstream inflammasome activity analyses supports a robust functional link between miR-223 upregulation and attenuated NLRP3-driven inflammation. Recognizing this as a limitation, the plan is to address it in future work by employing genetic editing strategies, such as CRISPR/Cas9 or AAV-mediated modulation, further to substantiate the mechanistic role of miR-223 in HN pathogenesis.

This study offers mechanistic insights into the renoprotective actions of YPSXZJDD *via* the miR-223/NLRP3/Caspase-1 axis, positioning miR-223 as both an effector and a regulatory fulcrum. These findings underscore the therapeutic potential of targeting microRNA-mediated inflammasome regulation and reinforce the translational significance of traditional Chinese medicine formulations in managing HN and associated inflammatory renal conditions.

## CONCLUSION

Through an integrated approach combining UHPLC-Q Exactive Orbitrap-MS, network pharmacology, and experimental validation, this study identifies Catalpol and Tanshinone IIA as the principal active components of YPSXZJDD, with both compounds exhibiting protective effects in HN. These effects are mechanistically linked to the upregulation of miR-223, which directly inhibits NLRP3 inflammasome activation, thereby mitigating renal epithelial cell apoptosis and inflammation. The observed reversal of therapeutic benefits upon miR-223 inhibition *in vivo* further substantiates the pivotal role of the miR-223/NLRP3/Caspase-1 axis in YPSXZJDD’s renoprotective action. Collectively, these findings support the rationale for targeting this molecular pathway in future HN treatment strategies and underscore the translational relevance of traditional Chinese medicine-based interventions in inflammatory kidney disorders.

## LIMITATIONS

While this study provides valuable insights into the potential of YPSXZJDD for treating hyperuricemic nephropathy, there are still several areas that deserve further exploration. For instance, although it is observed that YPSXZJDD upregulates miR-223 and downregulates the NLRP3/Caspase-1 pathway, the exact upstream signals that lead to miR-223 activation remain to be clarified. Moreover, although the role of miR-223 was demonstrated using inhibitor-based assays, direct genetic modulation (*e.g.*, overexpression or knockdown) of miR-223 was not performed to confirm its causal regulatory effect on NLRP3. Similarly, rescue experiments combining miR-223 inhibition with NLRP3 inhibitors (such as MCC950) were not conducted, which limits the mechanistic clarity of the miR-223/NLRP3 axis. These approaches should be prioritized in future studies to strengthen causal inference. Additionally, this animal model, while useful, may not fully replicate the complex and variable nature of human disease, which suggests that more diverse models and larger sample sizes would strengthen future findings. YPSXZJDD is a multi-herbal formula, and while the focus was on Catalpol and Tanshinone IIA as the key components, the potential contribution of other herbs in the recipe—and their synergistic interactions—should not be overlooked. Also, the investigation was centered on one microRNA, miR-223, which means other potentially important regulatory molecules might have been missed. Looking ahead, it would be helpful to conduct more comprehensive studies, perhaps incorporating broader RNA or proteomic analyses, to get a fuller picture of the mechanisms involved. Clinical validation in patients will also be an important step to confirm these findings and assess real-world safety and efficacy.

## Figures and Tables

**Table 1:** YPSXZJDD active ingredient screening.

Number	Metabolite	Formula	Retention Time(min)	Mode	Relative Abundance Value	OB(%)	DL
1	Stachyose	C_24_H_42_O_21_	0.766416667	neg	1.060488662	3.25	0.59
2	Engeletin	C_21_H_22_O_10_	6.297783333	neg	2.540314899	2.65	0.7
3	Cornuside	C_24_H_30_O_14_	6.0845	neg	1.293080138	2.61	0.71
4	Citrate	C_6_H_8_O_7_	1.206316667	neg	2.126847238	67.61	0.07
5	trans-5-Hydroxyferulic acid	C_10_H_10_O_5_	2.483983333	neg	1.028193236	59.99	0.07
6	Taxifolin 7-rhamnoside	C_21_H_22_O_11_	5.938833333	neg	4.824386935	57.84	0.27
7	Catalpol	C_15_H_22_O_10_	7.91555	pos	6.276034909	5.07	0.44
8	D-Proline	C_5_H_9_NO_2_	0.816316667	pos	2.460115173	63.39	0.06
9	Tanshinone IIA	C_19_H_18_O_3_	12.66613333	pos	8.202708135	49.89	0.4
10	Taxifolin	C_15_H_12_O_7_	6.12955	pos	1.045973911	57.84	0.27
11	D-Leucine	C_6_H_13_NO_2_	1.0652	pos	1.125854099	55.1	0.01
12	Nystose	C_24_H_42_O_21_	0.763616667	pos	1.105327147	3.95	0.57

## Data Availability

The data and supportive information are available within the article.

## LIST OF ABBREVIATIONS

HN: Hyperuricemic Nephropathy
CKD: Chronic Kidney Disease
YPSXZJDD: Yipishen Xiezhuo Jiedu Decoction
miR-223: microRNA-223
NLRP3: NOD-like receptor protein 3
Caspase-1: Caspase-1
GSDMD: Gasdermin D
EMT: Epithelial-to-Mesenchymal Transition
RAS: Renin–Angiotensin System
ROS: Reactive Oxygen Species
TGF-β1: Transforming Growth Factor Beta 1
SMAD3: Mothers Against Decapentaplegic Homolog 3
STAT3: Signal Transducer and Activator of Transcription 3
UHPLC-Q Exactive Orbitrap-MS: Ultra-High-Performance Liquid Chromatography Coupled with Quadrupole Exactive Orbitrap Mass Spectrometry
PPI: Protein–Protein Interaction
GO: Gene Ontology
KEGG: Kyoto Encyclopedia of Genes and Genomes
ELISA: Enzyme-Linked Immunosorbent Assay
qRT-PCR: Quantitative Real-Time Polymerase Chain Reaction
IHC: Immunohistochemistry
CCK-8: Cell Counting Kit-8
FBS: Fetal Bovine Serum
DMEM/F12: Dulbecco’s Modified Eagle Medium/Nutrient Mixture F-12
FITC: Fluorescein Isothiocyanate
PVDF: Polyvinylidene Difluoride
SDS-PAGE: Sodium Dodecyl Sulfate Polyacrylamide Gel Electrophoresis
DAB: Diaminobenzidine
ECL: Enhanced Chemiluminescence
FEUA: Fractional Excretion of Uric Acid
SUA: Serum Uric Acid
SCr: Serum Creatinine
BUN: Blood Urea Nitrogen
UUA: Urinary Uric Acid
UCr: Urinary Creatinine
